# *Caenorhabditis elegans* BAH-1 Is a DUF23 Protein Expressed in Seam Cells and Required for Microbial Biofilm Binding to the Cuticle

**DOI:** 10.1371/journal.pone.0006741

**Published:** 2009-08-25

**Authors:** Kevin Drace, Stephanie McLaughlin, Creg Darby

**Affiliations:** 1 Department of Cell and Tissue Biology, Program in Microbial Pathogenesis and Host Defense, University of California San Francisco, San Francisco, California, United States of America; 2 Department of Biology, Mercer University, Macon, Georgia, United States of America; Louisiana State University, United States of America

## Abstract

The cuticle of *Caenorhabditis elegans*, a complex, multi-layered extracellular matrix, is a major interface between the animal and its environment. Biofilms produced by the bacterial genus *Yersinia* attach to the cuticle of the worm, providing an assay for surface characteristics. A *C. elegans* gene required for biofilm attachment, *bah-1*, encodes a protein containing the domain of unknown function DUF23. The DUF23 domain is found in 61 predicted proteins in *C. elegans*, which can be divided into three distinct phylogenetic clades. *bah-1* is expressed in seam cells, which are among the hypodermal cells that synthesize the cuticle, and is regulated by a TGF-β signaling pathway.

## Introduction

The cuticle of the nematode *Caenorhabditis elegans* is vital for proper morphology, locomotion, and protection from pathogens and environmental stresses [1]. After the nematode hatches, this multi-layered, dynamic extracellular matrix is synthesized four times, with new cuticle replacing old at each molt [2]. The cuticle basal layer is attached to the hypodermal cells [1], and distal to it are the medial and cortical layers. These three layers, composed primarily of cross-linked collagens and cuticlins, provide structure, integrity and flexibility [2], [3]. The next layer, the epicuticle, contains lipids and perhaps proteins, but these have not been analyzed in detail [4], [5]. The most distal layer, the surface coat, is also poorly characterized. It can be visualized in transmission electron microscopy with cationized ferritin particles, suggesting that it is anionic [2], and indirect evidence suggests that the surface coat contains O-linked glycoproteins [6]. In the parasitic nematode *Toxocara canis* the surface coat is labile, requiring only ethanol for extraction [7], but this has not been examined for *C. elegans*.

Several phenotypes have been used in genetic studies of *C. elegans* surface determination. Antibody- and lectin-binding aberrations were used to isolate *srf* mutants [6], [8], [9], [10], [11]. Resistance to the nematode-specific pathogen *Microbacterium nematophilum* yielded numerous novel *bus* (bacterially unswollen) genes as well as alleles of *srf* genes. The *bus* study also described an assay for cuticle integrity using sensitivity to alkaline-hypochlorite treatment [12]. The causative agent of bubonic plague, *Yersinia pestis*, and its close relative, *Yersinia pseudotuberculosis*, form biofilms on the surface of the *C. elegans* head [13], [14], [15]. A screen for mutants resistant to biofilm attachment produced *bus* and *srf* alleles and also identified three novel *bah* (biofilm absent on head) genes: *bah-1*, *bah-2* and *bah-3* [16].

We now describe the cloning and initial characterization of *bah-1*, which encodes a member of a previously undescribed protein family found in nematodes, insects, plants and some non-mammalian vertebrates.

## Materials and Methods

### *C. elegans* strains

The wild-type strain N2 Bristol and JR667 *(wIs51)* SCM::GFP were used as were the following mutant genotypes of N2 Bristol; chromosomes are indicated by Roman numerals: (I) *dpy-5(e61), daf-16(mgDf47), unc-101(m1), daf-8(e1393),* (II) *daf-5(e1386), rrf-3(pk1426),* (III) *daf-2(e1370), daf-4(m63), daf-7(e1372),* (IV) *daf-1(m40), daf-14(m77),* (V) *him-5(e1467),* (X) *daf-3(e1376), daf-12(m25), lin-15(n765).*

### Biofilm formation on *C. elegans*

Biofilm assays were performed as previously described [14]. Briefly, adult worms were placed on lawns of *Y. pestis* or *Y. pseudotuberculosis* at room temperature and the presence of biofilm scored after 4 hours. For the analysis of the TGF-β pathway, all nematodes were grown at 15° C and assayed as adults at room temperature.

### Screen for additional *bah-1* alleles

Strain N2 was mutagenized with ethyl methanesulfonate (EMS) as described [17]. F_2_ animals resistant to biofilm accumulation were isolated as previously described [16]. New alleles were confirmed by complementation test against *bah-1(br1)*. In addition, a non-complementation screen was performed. Strain CB1467 *him-5(e1467)* was mutagenized with EMS and F_1_ male progeny mated to *C. elegans* strain DC1065 *dpy-5(e61) bah-1(br1) unc-101(m1)*. F_1_ progeny were treated with alkaline hypochlorite to release their eggs, which were washed in M9 buffer and placed onto lawns of *Y. pseudotuberculosis* strain YPIII. Bah non-Dpy non-Unc worms were selfed for two generations to recover a strain homozygous for a new *bah-1* allele. From approximately 9000 mutagenized genomes, one additional allele of *bah-1* was isolated, *br12*.

### Transgenic lines

Transgenic strains were produced by injecting yeast artificial chromosome (YAC), cosmid, or plasmid DNA into the gonad of young adult hermaphrodites as described [17]; for YAC experiments, we used total yeast DNA from strains carrying a YAC. Two transformation markers, *lin-15* and *rol-6*, were used. A wild-type *lin-15* (pbLH98) construct was injected, at approximately 60 ng/µl marker DNA and approximately 100–150 ng/µl test DNA, to transform strain DC1077 *bah-1(br1)* I; *lin-15(n765ts)* X. Stable F_2_ Lin+ lines were obtained and tested for rescue of the Bah phenotype on *Yersinia pseudotuberculosis* lawns. For the GFP expression construct, 100 ng/µl of *rol-6(su1006)* (pRF4), a dominant marker, was injected into wild-type *C. elegans* along with approximately 100 ng/µl pCBD156 and lines established from Rol progeny. For the epitope-tagged localization construct, plasmid pCBD219 was injected into DC1077 along with the *lin-15* marker as described above.

### Transgenic rescue

*bah-1* was previously mapped to a region deleted by the deficiency *hDf17* [16]. YAC clones Y53A2, Y47H9, Y53C10, and Y47H10 span this region. After complementation with Y53A2, cosmids spanning this YAC were obtained: T02A8, M04D5, C27D12, C27C7, ZK1025, K08C9, F32B4, F15D3, and C17D12. After cosmid ZK1025 complemented the Bah phenotype, single gene rescue was tested with a PCR-derived clone specific to the genes ZK1025.3, ZK1025.4, and ZK1025.7. All cosmid and YAC clones were obtained from the Sanger Institute (Cambridge, UK). To sequence mutant alleles, genomic DNA was harvested and PCR products corresponding to ZK1025.7 were analyzed for mutations.

### Plasmid construction

For rescue experiments with full length *bah-1*, a PCR fragment including *bah-1* and approximately 2 kb upstream, amplified with primers 5′-CGAACCTTAACTGGGGAGTAC and 5′-GACGATGATACGTGTACC, was cloned into pCR2.1-TOPO vector (Invitrogen, San Diego, CA), making the plasmid pCBD154. For expression analysis 2 kb upstream of *bah-1* was PCR amplified with primers 5′-CTGCAGGCGAACCTTAACTGGGGAGTAC and 5′-GGATCCTGTAATCAATATATGCCTTG that added restriction sites PstI and BamHI, respectively. The PCR product was ligated into vector pPD95.81 (Addgene plasmid 1497) to make pCBD156. For the 3×hemagglutinin (HA) tagged *bah-1* construct, full-length *bah-1* along with the 2 kb upstream was obtained with PCR primers 5′-GCCACTAGTCGAACCTTAACTGGGGAG and 5′-GCTCTGCAGGACGATGATACGTGTAC that introduced the restriction sites SpeI and PstI, respectively. The product was ligated into the vector pKH3 [18] to make pCBD219.

### Phylogenetic analysis of *C. elegans* DUF23

Phylogenetic analyses and construction was accomplished using MEGA4 [19]. Cladistic analysis used the maximum parsimony method [20] and the bootstrap consensus tree was inferred from 10,000 replicates. The Close-Neighbor-Interchange algorithm [21] was used to obtain the initial trees with the random addition of 10 replicates sequences.

### RNA interference (RNAi)

RNAi was performed using constructs from the Ahringer RNAi feeding library [22]. Briefly, an overnight culture was diluted and grown to exponential phase before the addition of isopropyl β-D-1-thiogalactopyranoside (IPTG) to induce double-stranded RNA production for approximately 2.5 hours at 37° C. Cultures were spiked with additional IPTG and used to seed Nematode Growth Medium (NGM) plates [17]. The next day L1/L2 stage *rrf-3(pk1426)* animals (hypersensitive to RNAi) were placed on these plates and incubated at 20° C for two days. Gravid adults from these plates were transferred to new NGM plates freshly seeded with the same RNAi construct induced as before. On the third day the F_1_ progeny were tested for cuticle defects.

### Immunofluorescence

*C. elegans* strain DC1154 carrying the construct pCBD219 was grown at 25° and transgenic worms identified by a Lin+ phenotype. L4 and early adult stage worms were fixed in 1% formaldehyde as described [17]. Incubation with anti-HA mouse monoclonal antibody 16B12 conjugated to Alexa Fluor 488 (Molecular Probes, Eugene OR) was carried out overnight at 4° at a 1∶25 dilution. Immunolocalization was carried as described using phosphate buffered saline (pH 8.0) containing 0.1% Tween 20 and 5% bovine serum albumin as a blocking agent [17]. Fluorescence was visualized using a Zeiss Axioplan 2 microscope.

## Results

### *bah-1* animals are resistant to biofilm attachment

**Figure 1:**
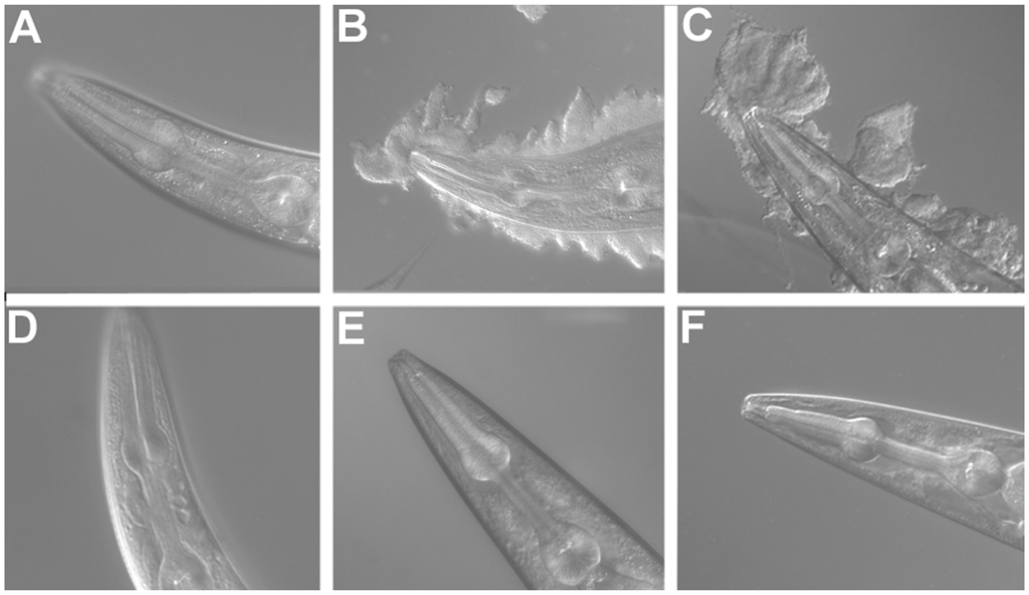
*bah-1* worms are resistant to biofilm attachment. (A–C) Wild-type *C. elegans* exposed to (A) *E. coli*, (B) *Y. pestis*, and (C) *Y. pseudotuberculosis.* (D–F) *bah-1* worms exposed to (D) *E. coli*, (E) *Y. pestis*, and (F) *Y. pseudotuberculosis*.

Biofilms produced by the bacterial species *Y. pestis* and *Y. pseudotuberculosis* attach to and accumulate on the head of *C. elegans* (Figure 1B and 1C). The biofilms physically block feeding, which for larval worms results in developmental arrest [23]. A screen for *C. elegans* mutants with altered biofilm binding identified three novel genes, including *bah-1* [16]. No biofilm attaches to *bah-1* animals in adult (Figure 1) or larval (not shown) stages, and their development is identical whether fed *E. coli* or *Yersinia*.

### Molecular identification of *bah-1*

The genetic deficiency *hDf17* failed to complement *bah-1* [16], narrowing the interval containing *bah-1* to approximately 4 map units on the right arm of chromosome I. YACs spanning this interval were injected into *bah-1* animals and transgenic lines established for each clone. YAC Y53A2 complemented the Bah phenotype, further narrowing the interval to approximately 350 kb. Next, overlapping cosmid clones spanning the Y53A2 region were injected, and rescue obtained with ZK1025, which contains nine genes. Finally, rescue was achieved with a single full-length gene on this cosmid, ZK1025.7 (Figure S1). Sequencing of seven *bah-1* mutant alleles confirmed that ZK1025.7 is *bah-1* (Table 1). An allele generated by the *C. elegans* Gene Knockout Consortium, *ok2197,* is a deletion that removes most of the coding region and is therefore presumptively null.

**Table 1:** Location and consequence of *bah-1* mutations.

Allele	Location^a^	Mutation	Consequence
*br1*	1332	a to t	E268V
*br12*	965	g to a	premature stop
*br21*	965	g to a	premature stop
*br22*	1657	g to a	aberrant splice junction
*br29*	1388	a to t	H279L
	1486	g to a	G312E
*br42*	1284	c to t	H245Y
*ok2197*	319–2060	deletion	incomplete transcript

a Distance from start ATG in base pairs.

### *bah-1* belongs to a large *C. elegans* gene family

The predicted *bah-1* mRNA (GenBank CAA18365) encodes a 461-amino acid, 53 kDa predicted protein. Amino acid residues 1–215 and 232–461 are confirmed by six expressed sequence tags (ESTs) in the Nematode Expression Pattern Database, National Institute of Genetics, Japan (clones yk841d05, yk1713e02, yk759d11, yk838c01, yk1505d06, yk842d05). No splice variants are predicted by these ESTs. We sequenced the cDNA clone yk1713e02 to confirm that residues 216–231 are expressed (data not shown). The SignalP and PSORT algorithms both predict a cleavable signal peptide, suggesting extracellular localization [24], [25]. The protein contains eight N-glycosylation motifs (Asn-Xaa-Ser/Thr, where Xaa is any amino acid except proline).

BAH-1 contains the domain of unknown function DUF23, comprising 267 amino acids, and has no other domains in the PFAM database [26]. DUF23 is found in 60 additional predicted *C. elegans* proteins. No mutations in any of the corresponding genes have been reported, although a genome-wide RNA interference (RNAi) screen found a molting defect for a single DUF23 gene, Y47D3B.1 [27]. There are no observable molting defects produced by any *bah-1* mutations, including the *ok2197* large deletion. Phylogenetic analysis (see below) indicates that Y47D3B.1 is an outlier within the DUF23 family, and unlike BAH-1 and most other *C. elegans* DUF23 proteins, it lacks a predicted signal sequence.

**Figure 2:**
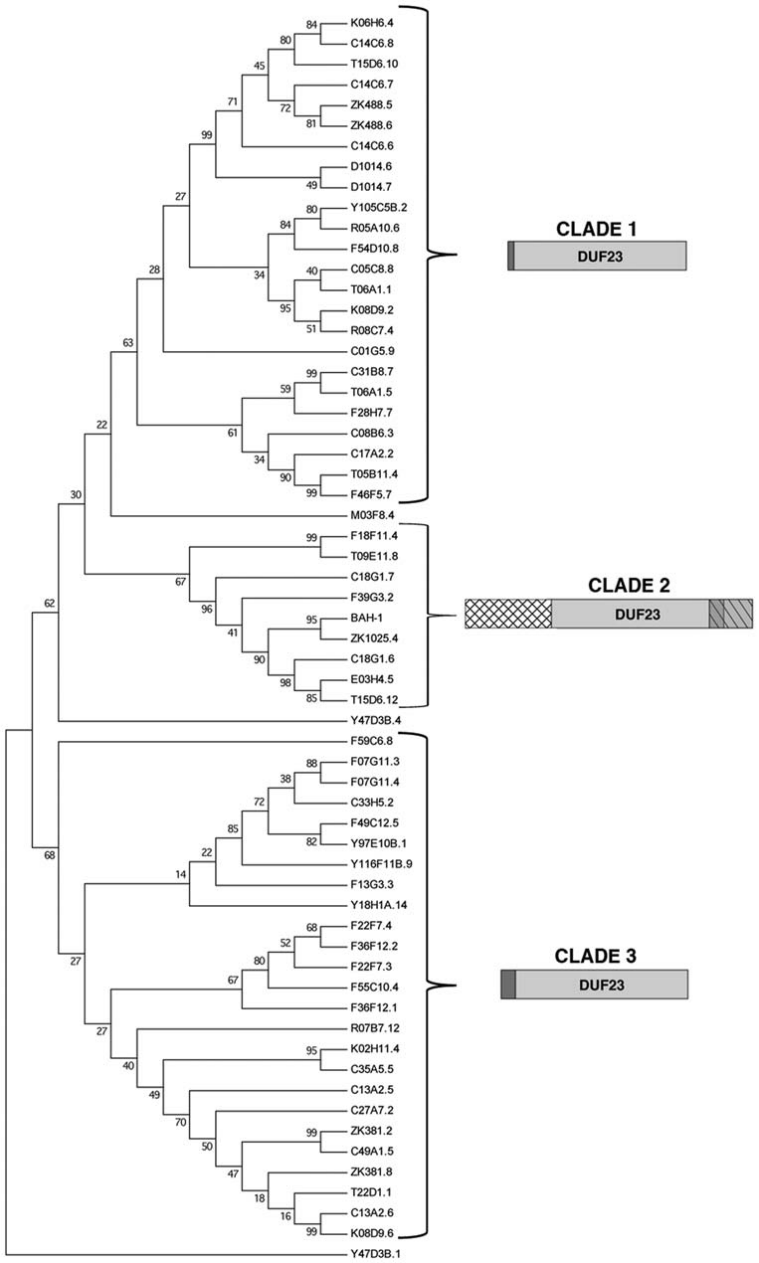
Phylogenetic relationships of 61 *C. elegans* DUF23 proteins determined by maximum parsimony. The bootstrap consensus tree shown is inferred from 10,000 replicates. Bootstrap values shown next to the branches were used to divide the family into three clades based on characteristic motif arrangements diagramed to the right of each clade. The DUF23 domain is shaded in light gray while conserved motifs found within each clade are distinguished by darker gray or a crosshatching pattern. The previously annotated DUF23 genes C13A2.1, C13A2.11, C13A2.2, D1014.5, K08D9.5, and T13H10.2 were eliminated from consideration because they lack the conserved cysteines and acidic amino acid motif, while C27C7.2 and ZK1025.5 were eliminated due to a high probability of being pseudogenes.

We analyzed the *C. elegans* DUF23 family using a maximum parsimony phylogenetic method. Most proteins fell into one of three distinct clades, each of which has unique features (Figure 2). Clade 1 proteins, which cluster away from other DUF23 proteins with a 63% bootstrap value, have a common motif consisting of approximately 10 amino acids directly N-terminal to the DUF23 domain. Clade 2, of which BAH-1 is a member, is characterized by two motifs. The first, approximately 150 residues, lies N-terminal to DUF23, while the second begins within the C-terminal region of DUF23 and continues beyond it for approximately 75 residues. The portion of this second motif that lies within DUF23 is more highly conserved within clade 2 than in the other two clades. Lastly, clade 3 proteins (68% bootstrap) have a common motif of approximately 25 amino acids N-terminal to DUF23. A number of proteins do not cluster with any of these clades, including the one implicated in molting, Y47D3B.1 (Figure 2).

**Figure 3:**
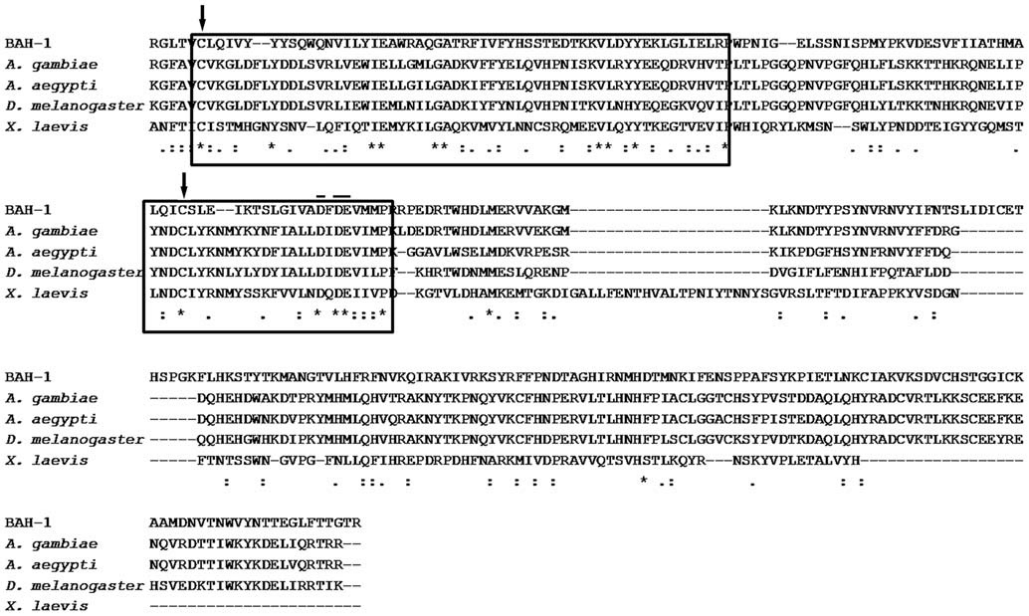
CLUSTAL alignment of the DUF23 domains of BAH-1 and its closest relatives in three insect species and *X. laevis*. Boxed regions are the most highly conserved within DUF23 proteins. Arrows show cysteine residues with conserved spacing. Bars designate conserved acidic motif.

The DUF23 domain is found in insects, plants, and some bacteria. It is also in two vertebrates, the frog *Xenopus laevis* and the fish *Tetraodon nigroviridis*, but absent from all sequenced mammalian genomes. Genes encoding DUF23 proteins are far more abundant in free-living nematode genomes than in the genomes of other taxa. The fruit fly *Drosophila melanogaster* has five predicted DUF23 proteins while the mosquitoes *Anopheles gambia* and *Aedes aegypti* each have three. Figure 3 shows the alignment of DUF23 domains of BAH-1, insects and *X. laevis*. DUF23 domains of other species are more similar to BAH-1 and other members of clade 2 than they are to the *C. elegans* DUF23s in the other clades.

Across the entire family, the most highly conserved DUF23 features are a small acidic motif and two cysteines with conserved spacing (Figure 3). The *br1* mutation results in a glutamic acid to valine substitution in the acidic motif. The Bah phenotype of *br1* animals is identical to that of worms carrying the presumptively null *ok2197* deletion, suggesting that *br1* is also null and therefore that the acidic motif is essential to protein function. All other *bah-1* mutations analyzed also disrupt the DUF23 domain.

Six *C. elegans* proteins annotated as DUF23 in databases (13, 29) lack the conserved cysteines, lack the acidic domain or have low overall similarity to the majority of family members, and we consider these to be misannotated (Figure 2).

### *bah-1* is expressed in seam cells

**Figure 4:**
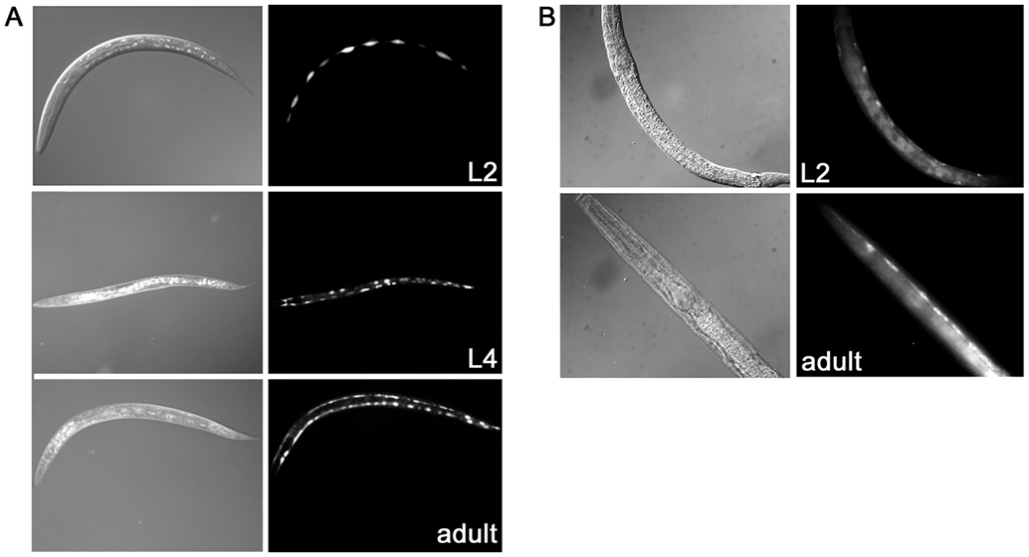
*bah-1* expression in seam cells. (A) Expression of promoter-GFP fusion (Experimental Methods) in L2, L4 and early adult. (B) Immunofluorescence detection of epitope-tagged BAH-1 in two representative worms.

We fused the putative *bah-1* promoter to a green fluorescent protein (GFP) gene and observed expression in the lateral seam cells (Figure 4A), a subset of the hypodermis. We obtained a strain with an integrated seam-cell specific promoter fused to GFP, JR667 *(wIs51)* SCM::GFP, and compared the signals to our *bah-1* promoter-GFP signals; at all stages the expression patterns were the same (data not shown). Expression was detectable beginning in the early larval stages (L1/L2) and continuing into adulthood, consistent with the temporal expression reported in a functional genomics study [28]. To determine protein localization we generated transgenic worms expressing full-length BAH-1 with an added C-terminal HA tag. Expression of this construct rescued a *bah-1* mutation, indicating that the modification did not disrupt function. In fixed, permeabilized worms, BAH-1 was detected in association with hypodermal cells, including the seam cells, consistent with the promoter fusion data (Figure 4B). We were unable to detect BAH-1 on the surface of unpermeabilized worms with antibody against the HA tag.

### *bah-1* is regulated by the TGF-β signaling pathway

A TGF-β pathway regulates *C. elegans* entry into the alternative developmental stage known as the dauer larva, which the worm uses to survive under certain harsh conditions. TGF-β signaling inhibits the transcriptional regulators DAF-3 and DAF-5 that are required for dauer formation. Mutants with defects in the TGF-β ligand gene *daf-7*, the receptor genes *daf-1* and *daf-4*, and the downstream SMAD signaling genes *daf-8* and *daf-14* constitutively make dauers at 25°C under non-inducing conditions (Daf-c phenotype). Conversely, *daf-3* and *daf-5* mutants are dauer defective under inducing conditions (Daf-d) [29].

A global transcriptional analysis showed that *bah-1* expression was down-regulated an average of 19-fold in the absence of *daf-7, daf-8* or *daf-14* [30]. We confirmed that *daf-7* animals have decreased *bah-1* expression using semiquantitative RT-PCR (data not shown). The down-regulation prompted the hypothesis that mutants with TGF-β signaling defects would be Bah, and indeed this was previously reported for an unspecified *daf-1* allele [15].

**Figure 5:**
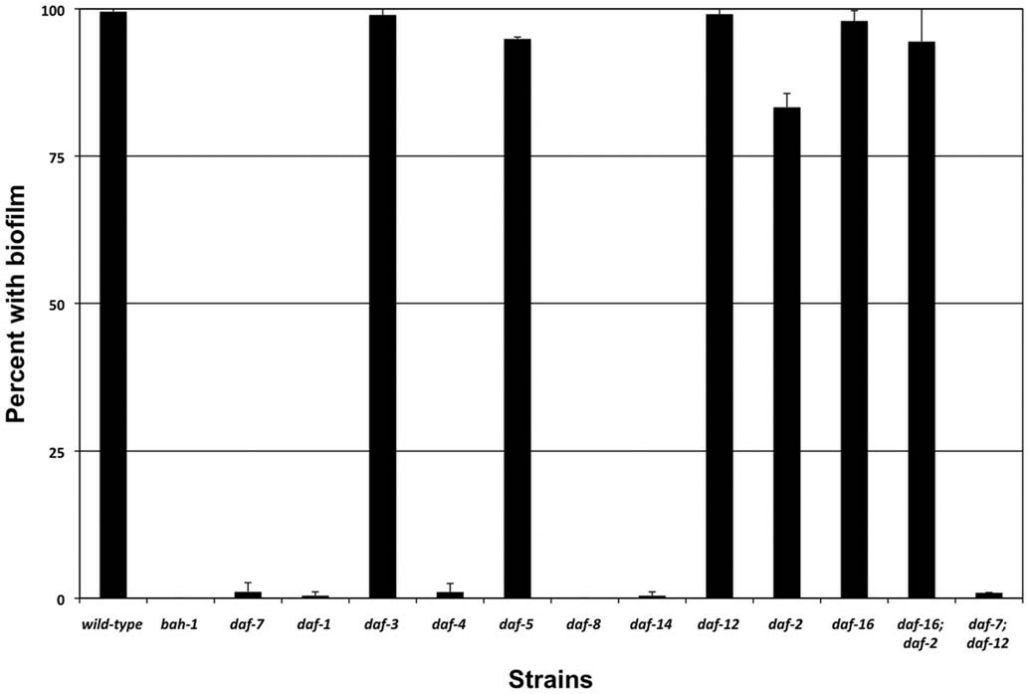
Biofilm attachment phenotypes of *bah-1* null, dauer defective, and dauer constitutive mutants. Biofilm attached after 4 hours of exposure to *Y. pseudotuberculosis*. Data are mean±S.D. of two independent trials, with a minimum of 82 animals from each genotype per trial.

We previously showed that biofilms do not form on dauers [16]. To examine the biofilm phenotypes of non-dauer animals with defects in TGF-β signaling, mutants were grown at 15°C, because at this temperature even worms with null mutations in Daf-c genes do not become dauers [31]. We tested *daf-7(e1372), daf-1(m40), daf-4(m63), daf-8(e1393)* and *daf-14(m77),* each of which has a strong Daf-c phenotype [32], [33] and one of which, *daf-14(m77)*, is probably null [31]. Each mutation produced a complete or nearly complete Bah phenotype, with no more than 2% of animals acquiring biofilms (Figure 5). The results are consistent with the *bah-1* down-regulation observed in *daf-7, daf-8* or *daf-14* mutants [30], but do not exclude the possibility that TGF-β regulates other genes required for biofilm attachment. Mutants with defects in the Daf-d genes *daf-3* and *daf-5* were not Bah (Figure 5), indicating that TGF-β positive regulation of *bah-1* is not mediated through DAF-3 or DAF-5.

Dauer formation is also regulated by insulin/IGF-1 signaling, and mutations in the receptor gene of this pathway, *daf-2*, produce a Daf-c phenotype at 25°C. DAF-2 represses dauer formation by inhibiting the FOXO transcription factor DAF-16. Both *daf-16* null animals and *daf-16; daf-2* double mutants are Daf-d.

When grown at 15°C to avoid dauer formation, about 85% of *daf-2* adults acquired biofilms (Figure 5). This weak Bah phenotype is in contrast to the nearly complete absence of biofilms on TGF-β pathway mutants. *daf-16* null and *daf-16; daf-2* animals had the wild-type biofilm phenotype (Figure 5).

A steroid hormone receptor required for dauer formation, DAF-12, is downstream of both TGF-β and insulin-like pathways [32], [34]. *daf-12* mutants are not Bah, while a *daf-7; daf-12* mutant was biofilm defective, indicating that TGF-β positive regulation is not mediated through DAF-12.

### Disruption of other DUF23 genes does not produce the Bah phenotype

Using RNAi we tested 21 other *C. elegans* DUF23 proteins for aberrant biofilm attachment (Table S1). Three criteria were used to choose those tested: they are in the same clade as BAH-1 (Figure 2), the developmental timing of their expression is similar to that of BAH-1 [28], or they are regulated by the TGF-β pathway [30]. Only RNAi on *bah-1* and ZK1025.4 resulted in a Bah phenotype (Table S1). ZK1025.4 is the *C. elegans* DUF23 most similar to *bah-1*, and there is sufficient DNA sequence identity to predict cross-reaction of RNAi constructs. RT-PCR analysis showed that ZK1025.4 RNAi indeed reduced *bah-1* expression, suggesting that the Bah phenotype observed was an off-target effect (data not shown). Attempts to reduce ZK1025.4 expression by RNAi without affecting *bah-1* expression were unsuccessful.

## Discussion

### Biofilm attachment reveals a role for a DUF23 protein in determining *C. elegans* surface characteristics

The composition, structure and function of the *C. elegans* exterior surface are poorly understood. Biochemical analysis, using sequential extractions of increasing harshness, provided evidence for a heterodimeric protein complex distal to the collagen layers, probably in the epicuticle [4]. The identities of the two proteins were not determined. Genetic analysis, using antibodies and lectins to probe the surface, identified genes *srf-2*, *srf-3* and *srf-5*, which appear to have primary defects in surface properties [9], [10]. Here, too, the surface defects have not been determined at the molecular level. *srf-3* encodes a nucleotide sugar transporter, a protein that mediates intracellular trafficking of the glycosylation substrate UDP-galactose [35]. A biochemical analysis confirmed that *srf-3* mutants are globally deficient in galactosylation [36], but the specific glycoconjugates that are absent or aberrant are not known. Infection with *M. nematophilum* identified additional glycosylation genes that affect the surface: *bus-17* [37], *bus-2, bus-4* and *bus-12* (J. Hodgkin, personal communication). Mutations in any of these four genes produce the Bah phenotype [16].

*Yersinia* sp. biofilms that bind the *C. elegans* exterior provide an additional assay for the nematode surface. Ultrastructural analysis of biofilm binding does not show any physical perturbation of the cuticle's subsurface collagen layers [13], and biofilm binding is normal when collagen genes, e.g. *dpy-5, dpy-9, dpy-17* and *rol-6*, are mutated (data not shown). Even *bli-6* mutants, whose cuticle becomes heavily blistered, bind biofilm normally (data not shown). In contrast, mutations in any of the glycosylation genes found to affect the surface (*bus-2, bus-4, bus-12, bus-17, srf-3*) produce the Bah phenotype, as do mutations in the uncloned surface-determining genes *srf-2* and *srf-5* [16]. Together, these observations indicate that biofilm attachment is to distal cuticle components, i.e. the surface coat and/or epicuticle. The Bah phenotypes of multiple glycosylation mutants suggest that the biofilm receptor either is itself a glycoconjugate or that its function requires at least one glycoconjugate.

Using the absence of biofilm phenotype (Bah) in a mutant screen, we identified three genes, *bah-1, bah-2* and *bah-3*, without other strong phenotypes [16]. In particular, they behave normally in response to *M. nematophilum* infection, suggesting that they are specific for the surface characteristics that mediate biofilm attachment. *bah-1* and *bah-2* cuticles are mildly sensitive to alkaline hypochlorite treatment, while *bah-3* mutants have normal integrity [16].

*bah-1* encodes one of the many *C. elegans* proteins containing a DUF23 domain. A fully penetrant Bah phenotype is produced by each of seven alleles, four of which *(br12, br21, br22* and *ok2197*) are likely null and three of which (*br1, br29* and *br42*) are missense mutations (Table 1). The *br1* mutation could be especially informative about DUF23 function because it disrupts the most highly conserved motif in the family (Figure 3).

No DUF23 genes have been identified in previous *C. elegans* mutant screens. A genome-wide RNAi screen found a molting defect for Y47D3B.1, but not for any other DUF23 protein [27]. Although Y47D3B.1 has the common cysteines and acidic motif of DUF23 domains, it is an outlier in the *C. elegans* family. It does not fall within one of the three clades in our phylogenetic analysis, and unlike BAH-1 and all other members of clade 2, it has no predicted signal sequence. There is no molting defect in *bah-1* mutants, including the presumptively null deletion allele *ok2197*, nor is there evidence that DUF23 proteins other than Y47D3B.1 function in molting. We conclude that *bah-1* does not participate in molting.

### BAH-1 expression in seam cells

The *C. elegans* cuticle is secreted by hypodermal cells. Seam cells, a hypodermal subset in bilateral rows along the length of the animal, participate in this process [27], [38]. BAH-1 is expressed in seam cells (Figure 4), and its temporal regulation correlates with that of collagen and cuticlin genes that encode the major cuticle components [1], [39]. One function of seam cells is to synthesize alae, which are lateral cuticle ridges that run the length of the animal directly over the seam cells in L1 larvae, dauer larvae, and adults [38]. Alae are not present in L2, L3 and L4 stage worms, but *Yersinia* sp. biofilms bind these stages [14], indicating that alae are not required for biofilm binding and that BAH-1 function is not alae-specific. Mutations in the gene *srf-3* also lead to aberrant surface phenotypes including resistance to biofilm attachment [35], [40]. Like *bah-1*, *srf-3* is expressed in lateral seam cells, further implicating these cells in the production of surface determinants and suggesting a role for these proteins in cuticle development.

The presence of a signal sequence, conserved cysteine spacing and N-glycosylation motifs together suggest that BAH-1 is extracellular, consistent with the possibility that it is part of the epicuticle or surface coat. However, we did not detect epitope-tagged BAH-1 on the surface of live, undisrupted worms. This suggests that BAH-1 remains cell-associated, but we cannot exclude the possibilities that it is within the extracellular matrix but not detected because of low abundance, covalent modification before or during secretion, masking of the epitope by other surface components, or loss during processing for staining.

### Regulation by TGF-β

*C. elegans* is capable of sensing its environment and adapting gene expression accordingly [41]. A well-studied example is sensing of environmental stress (scarce food, overcrowding and high temperature) and subsequent entry into the non-feeding, non-reproducing dauer stage [4], [38]. This developmental decision, made during the L1 stage, is regulated by both TGF-β and insulin/IGF-1 signaling pathways [29]. Under non-stress conditions, receptors in both pathways are activated, dauer formation is repressed, and reproductive development proceeds. When signaling is down-regulated or absent, the dauer pathway is activated.

Dauers differ from the corresponding normal larval stage (L3) in morphology, physiology and behavior. Of particular relevance for the current study, the dauer cuticle has a markedly different ultrastructure and, unlike any other stage, is resistant to 1% sodium dodecylsulfate [2]. Differences in cuticle collagen composition between dauers and non-dauers have been examined [42], but the surface components that are distal to collagen layers have not been determined for either dauers or non-dauers.

*Yersinia* biofilms do not bind dauers, but bind the surface of all non-dauer stages in a *bah-1*-dependent manner. A global transcriptional analysis showed that *bah-1* expression requires the TGF-β ligand gene *daf-7* and the SMAD genes *daf-8* and *daf-14* [30]. We confirmed that mutations in these genes, as well as the *daf-1* and *daf-4* receptor genes, produce a strong Bah phenotype (Figure 5). Because loss of *bah-1* is sufficient for Bah, our data do not address whether TGF-β has other targets affecting surface characteristics.

Evidence from another assay indicates that it does. A monoclonal antibody, M37, binds the surface of wild-type *C. elegans* first stage (L1) larvae but no other developmental stages. However, M37 aberrantly binds all non-dauer larval stages of some mutants, a phenotype known as constitutive larval display (Cld) [11]. Mutations in *daf-1, daf-4, daf-7, daf-8* and *daf-14* all produced a strong Cld phenotype when worms were grown under non-dauer-inducing conditions [43]. The antigen recognized by M37 is unknown, but it is present in *bah-1* mutants and therefore cannot be BAH-1 [16]. The Bah and Cld phenotypes of TGF-β mutants indicate that the surface properties of Daf-c mutants are aberrant, even when the worms are grown at 15° C and do not actually form dauers. The defect cannot be explained by the simple hypothesis that the non-dauer Daf-c mutants have surfaces identical to those of dauers, because M37 does not bind dauer larvae [11].

Like multiple TGF-β signaling mutants, *daf-2* mutants are strongly Daf-c. Unlike the TGF-β mutants, however, *daf-2* animals have only a weak Bah phenotype. Most *daf-2* adults acquired biofilms; only about 15% did not, and the defect was suppressed by a *daf-16* mutation (Figure 5). Also unlike TGF-β Daf-c mutants, *daf-2* animals do not have the Cld phenotype [11]. We could not determine the Bah phenotype of *daf-2* null mutations, because such mutations are lethal [44]. However, the *daf-2(e1370)* allele we tested has a fully penetrant Daf-c phenotype at 25° C [32] and a strong longevity phenotype [45], indicating that even strong *daf-2* mutations produce only a weak Bah phenotype.

These results suggest that during dauer formation, DAF-16 represses surface-determining genes, perhaps including *bah-1*, that are only required in non-dauer stages. In wild-type animals under reproductive growth conditions, repression would not occur because DAF-2 inhibits DAF-16. In *daf-2* mutants under these conditions, DAF-16 is aberrantly activated, leading to repression of surface-determining genes. This is plausible in light of findings that in *daf-2* mutants, dauer-stage genes are inappropriately expressed in non-dauers [46]; presumably inappropriate repression of non-dauer-stage genes also occurs.

Although the role of TGF-β in regulating dauer formation has been extensively investigated, the pathway's regulation of non-dauer characteristics has been little examined [47]. Taken as a whole, our analysis of dauer pathway regulatory mutants suggests that the TGF-β pathway is a major positive regulator of surface-determining genes such as *bah-1* that are expressed during reproductive growth. Unlike TGF-β activity in the dauer pathway, the surface gene regulation is not mediated by DAF-3, DAF-5 or DAF-12. Our results also indicate a possible subsidiary role for the insulin/IGF-1 pathway in repressing surface-determining genes during dauer formation.

### The DUF23 family in *C. elegans* and other organisms

*C. elegans* has 61 proteins containing DUF23, and the family is also large in the related species *Caenorhabditis remanei* (90 representatives) and *Caenorhabditis briggsae* (60 representatives). Accordingly, these proteins have representatives that fall into the same three phylogenetic clades as *C. elegans* [48], WormBase Release WS170). Interestingly, searches of the incomplete genomes of the human parasitic nematodes *Brugia malayi* and *Trichinella spiralis* and plant-parasitic nematodes *Meloidogyne hapla* and *Heterodera glycines* did not find DUF23 domains.

In our phylogenetic analysis we divided the *C. elegans* family into three clades (Figure 2). We found six *C. elegans* proteins that appeared to be misclassified, because they did not have the conserved acidic motif that is present in all others DUF23 proteins. Nevertheless, the number of DUF23 proteins in *C. elegans* and in related nematodes greatly exceeds that in any other sequenced organism. In the Pfam database (http://pfam.sanger.ac.uk) the most DUF23 proteins outside of nematodes is 13 in the mustard *Arabidopsis thaliana*. No more than five are in the genome of any animal that is not a nematode [26].

At least one DUF23 is present in every insect genome available in GenBank. This is significant because the biofilm system used to identify and characterize *bah-1* is a model for *Y. pestis* colonization of its vector, the flea [13], [23]. Formation of a biofilm in the flea digestive tract is essential for transmission of this pathogen [49]. The BAH-1 requirement for *C. elegans* biofilm attachment, coupled with the existence of DUF23 proteins in several sequenced insect genomes, suggests that it will be worthwhile to determine whether fleas express this domain at the site of biofilm binding and, if so, whether it has a role in biofilm adherence.

Bioinformatic analysis has grouped protein families such as DUF23 in larger, multi-family “clans” that are believed to be of common evolutionary origin [50]. DUF23 is assigned to clan GT-A, which contains multiple glycosyltransferase families as well as other types of carbohydrate-interacting proteins [51]. BAH-1 may directly interact with carbohydrates by binding to specific sugar residues or indirectly by participating in a pathway for the specific glycoconjugate production required for biofilm attachment to the surface of *C. elegans*. Regardless, a BAH-1 interaction with carbohydrates, is consistent with the finding that multiple genes known to function in glycosylation are required for biofilm adherence to *C. elegans*.

### Concluding remarks

The nature of the *C. elegans* surface and the manner in which it interacts with the environment are largely unknown. Using attachment of *Yersinia* sp. biofilms as a probe for the surface, we have found that *bah-1* plays a role in determining the nematode's surface characteristics. Neither *bah-1* nor any member of the large DUF23 family to which it belongs was identified by other mutant phenotypes, which demonstrates the utility of the biofilm phenotype in analyzing *C. elegans* surface characteristics. We have shown that BAH-1, possibly a secreted protein, is expressed in seam cells, a subset of the cuticle-synthesizing hypodermis. Obviously the evolved purpose of BAH-1 is not to mediate adherence of a deleterious microbial product. The normal function is not known, nor is the reason why *C. elegans* and related free-living nematodes have many more DUF23 proteins than organisms in other taxa. As a motile soil organism, *C. elegans* undoubtedly encounters a wide variety of environmental threats. Mutation in *bah-1* affect the worm's interaction with a microbial exopolysaccharide, and also cause a slight increase in the cuticle's sensitivity to harsh reagents. These findings prompt the hypothesis that DUF23 proteins function in maintaining cuticle integrity against environmental assaults.

## Supporting Information

Figure S1 *bah-1* cloning. (A) Chromosome I, the deficiency hfDf17, Yeast artificial chromosomes (YACs) and cosmid clones in approximate relative positions. Numbers indicate ratios of rescuing lines to total transgenic lines for each clone. “Genes” shows rescue results for PCR-derived single gene constructs. (B) Exon-intron structure and locations of mutations were obtained from full length cDNA sequences from the National Institute of Genetics, Mishima, Japan.
(9.68 MB TIF)

Table S1 (0.03 MB XLS)
