# Supplementary figures and images for: Caenorhabditis elegans BAH-1 Is a DUF23 Protein Expressed in Seam Cells and Required for Microbial Biofilm Binding to the Cuticle
Source: PLoS One. 2009 Aug 25;4(8):e6741. doi: 10.1371/journal.pone.0006741 (PMC2727005; doi:10.1371/journal.pone.0006741)

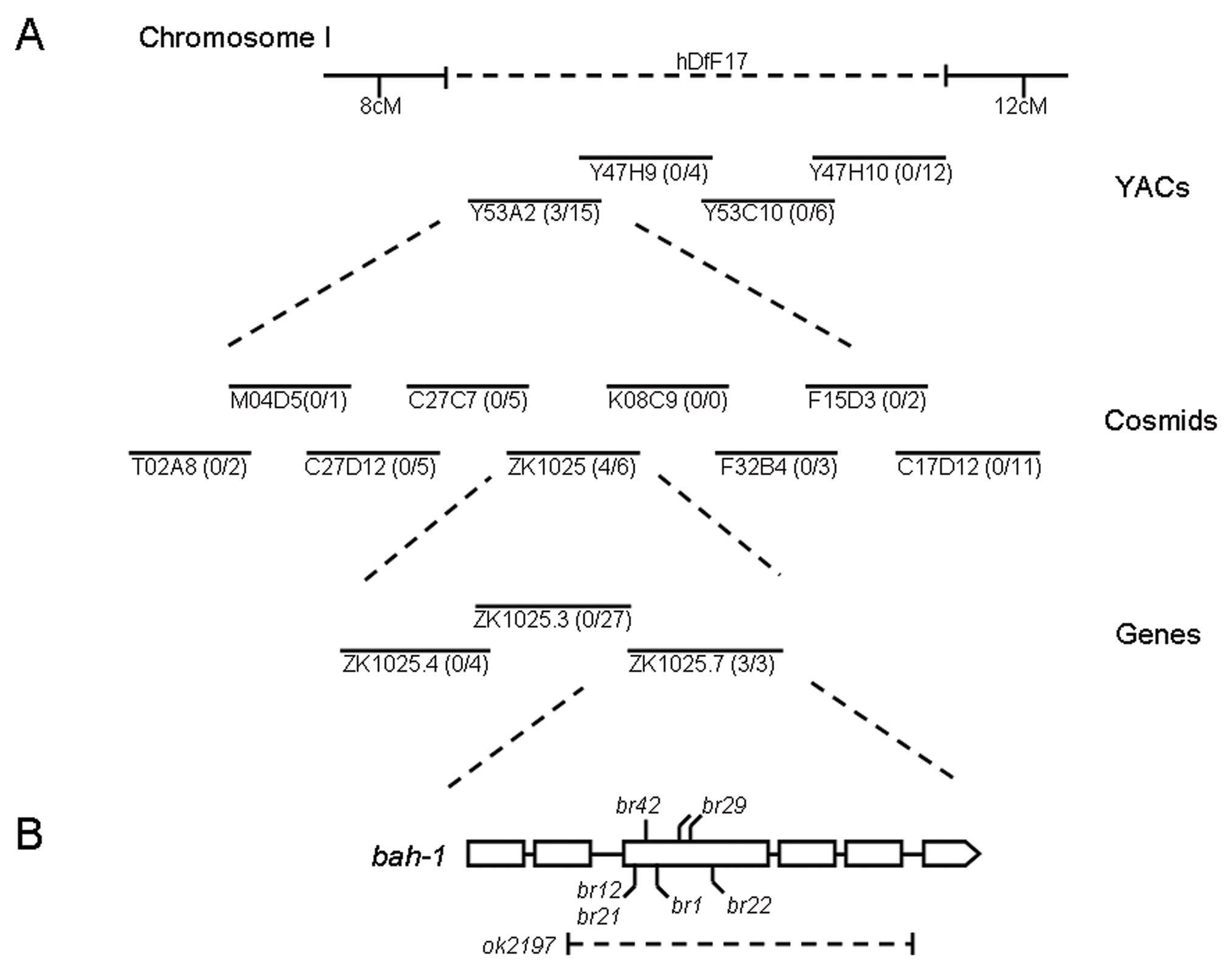

Supplement: Figure S1 — bah-1 cloning. (A) Chromosome I, the deficiency hfDf17, Yeast artificial chromosomes (YACs) and cosmid clones in approximate relative positions. Numbers indicate ratios of rescuing lines to total transgenic lines for each clone. “Genes” shows rescue results for PCR-derived single gene constructs. (B) Exon-intron structure and locations of mutations were obtained from full length cDNA sequences from the National Institute of Genetics, Mishima, Japan. (9.68 MB TIF) [file pone.0006741.s001.tif]
